# The F_0_F_1_-ATP Synthase Complex Contains Novel Subunits and Is Essential for Procyclic *Trypanosoma brucei*

**DOI:** 10.1371/journal.ppat.1000436

**Published:** 2009-05-15

**Authors:** Alena Zíková, Achim Schnaufer, Rachel A. Dalley, Aswini K. Panigrahi, Kenneth D. Stuart

**Affiliations:** Seattle Biomedical Research Institute, Seattle, Washington, United States of America; Stanford University, United States of America

## Abstract

The mitochondrial F_0_F_1_ ATP synthase is an essential multi-subunit protein complex in the vast majority of eukaryotes but little is known about its composition and role in *Trypanosoma brucei*, an early diverged eukaryotic pathogen. We purified the F_0_F_1_ ATP synthase by a combination of affinity purification, immunoprecipitation and blue-native gel electrophoresis and characterized its composition and function. We identified 22 proteins of which five are related to F_1_ subunits, three to F_0_ subunits, and 14 which have no obvious homology to proteins outside the kinetoplastids. RNAi silencing of expression of the F_1_ α subunit or either of the two novel proteins showed that they are each essential for the viability of procyclic (insect stage) cells and are important for the structural integrity of the F_0_F_1_-ATP synthase complex. We also observed a dramatic decrease in ATP production by oxidative phosphorylation after silencing expression of each of these proteins while substrate phosphorylation was not severely affected. Our procyclic *T. brucei* cells were sensitive to the ATP synthase inhibitor oligomycin even in the presence of glucose contrary to earlier reports. Hence, the two novel proteins appear essential for the structural organization of the functional complex and regulation of mitochondrial energy generation in these organisms is more complicated than previously thought.

## Author Summary

African trypanosomes (*Trypanosoma brucei* and related subspecies) are unicellular parasites that cause the devastating disease of African sleeping sickness in man and nagana in livestock. Both of these diseases are lethal, killing thousands of people each year and causing major economical complications in the developing world, thus affecting the lives of millions. Furthermore, available drugs are obsolete, difficult to administer and have many undesirable side-effects. Therefore, there is a reinvigorated effort to design new drugs against these parasites. From the pharmacological perspective, unique metabolic processes and protein complexes with singular structure, composition and essential function are of particular interest. One such remarkable protein complex is the mitochondrial F_0_F_1_-ATP synthase/ATPase. Here we show that F_0_F_1_-ATP synthase complex is essential for viability of procyclic *T. brucei* cells and it possesses unique and novel subunits. The three F_0_F_1_-ATP synthase subunits that were tested were shown to be crucial for the structural integrity of the F_0_F_1_-ATP synthase complex and its activities. The compositional and functional characterization of the F_0_F_1_-ATP synthase in *T. brucei* represents a major step towards deciphering the unique and essential properties of the respiratory chain of both an early diverged eukaryote and a lethal human parasite.

## Introduction

Trypanosomes and related kinetoplastids parasites are responsible for several serious infectious diseases of human and livestock worldwide. The few available drugs are difficult to administer, have severe side-effects, and suffer from increasing resistance [1]. For that reasons, improved drug therapy of kinetoplastid infections and the identification of new molecular targets are important goals.

*Trypanosoma brucei* has a complex life cycle alternating between a mammalian host and a blood-feeding insect vector, the tsetse fly. The procyclic insect stage (PF) lives in the insect midgut and feeds mainly on two amino acids, proline and threonine, which are converted into partially oxidized end products by so-called aerobic fermentation [2]. The single large branched mitochondrion of these PF cells is fully developed with many cristae, Krebs cycle enzymes, and abundant levels of mitochondrial (mt) F_0_F_1_-ATP synthase (respiratory complex V). It has a complete respiratory chain that oxidizes the reduced equivalents generated by amino acid metabolism and the glycolytic pathway and thus generates indispensable membrane potential [3].

The bloodstream form (BF) is well adapted to an environment with a constant level of blood glucose and energy requirements are met by an aerobic type of glycolysis where glucose is converted to pyruvate. The metabolic role of the single tubular BF mitochondrion is suppressed and the organelle lacks a functional respiratory chain and mt membrane potential involves the reverse function of the F_0_F_1_-ATP synthase: the complex hydrolyzes ATP produced by glycolysis to pump protons from the matrix to the inter-membrane space [4]–[7]. This reverse function of the F_0_F_1_-ATPase complex is indispensable for BF trypanosomes and an inhibitor specifically targeting the F_0_F_1_-ATPase activity would be expected to be lethal to trypanosomes, but not the host, which utilizes the conventional function of this complex to create ATP. Importantly, these inhibitors may be adapted from those already developed to prevent tissue damage caused by ischemic conditions in humans. Therefore, the trypanosomatid F_0_F_1_-ATPase is an attractive anti-trypanosomal drug target.

Generally, F_0_F_1_-ATPsynthase/ATPase is a ubiquitous enzyme comprised of two oligomeric components, F_0_ and F_1_, linked together by a central and a peripheral stalk [8],[9]. The hydrophilic domain F_1_ bears three catalytic sites and extends into the matrix. The hydrophobic domain F_0_ is membrane embedded and contains a proton channel. The prokaryotic enzyme, which represents the simplest form of the complex, appears to consist of five different protein subunits of F_1_ (α_3_β_3_γδε) and three subunits of F_0_ (*ab*_2_ *c*_10–12_) (multiple stoichiometry indicated in subscript). These subunits form the core of the F_0_F_1_ motor structure. The eukaryotic enzyme has homologous components, but also incorporates additional subunits involved in the structure and regulation of the complex (Table 1). With the exception of subunit ε and IF1, which bind to F_1_, the additional subunits A6L (subunit 8 in yeast), F6 (subunit *h* in yeast), *d*, *e*, *f*, *g* and oligomycin sensitivity-conferring protein (OSCP) are associated with the F_0_ proton channel or the peripheral stalk [10]. Additionally, the yeast enzyme contains supernumerary subunits *i* and *k* [11]–[13] and bovine complex contains additional subunits AGP and MLQ [14].

**Table 1:** F_0_F_1_-ATP synthase subunits nomenclature.

	Bacterial enzyme	Mitochondrial enzyme		
		*Saccharomyces cerevisiae*	*Bos taurus*	*Trypanosoma brucei* (*E*-value^a^)
F_1_	α	α	α	Tb927.7.7420/Tb927.7.7430 (5e-114)
	β	β	β	Tb927.3.1380 (5e-162)
	γ	γ	γ	Tb10.100.0070 (2e-13)
	ε	δ	δ	Tb927. 6.4990 (4e-13)
	-	ε	ε	Tb10.70.2155 (2e-4)
F_0_	δ	OSCP	OSCP	Tb10.6k15.2510 (0.0021)
	*a*	6	*a*	NCBI: AAA97428 (mt encoded)^b^
	*b*	9	*b*	Tb927.5.1710^b^
	*c*	4	*c*	Tb10.70.6340 (5e-8)
				Tb11.02.2950 (8e-08)
				Tb927.7.1470 (6e-08)
	-	8	A6L	-c
	-	*d*	*d*	-
	-	*f*	*f*	-
	-	*h*	F6	-
Associated proteins	-	IF_1_	IF_1_	-
	-	*g*	*g*	-
	-	*e*	*e*	-
	-	*i*	-	-
	-	*k*	-	-
	-	-	AGP	-
	-	-	MLQ	-

a *E*-values were obtained by BLAST search analysis using bovine ATP synthase subunits as subject.

b *T. brucei* homologues of subunits *a* and *b* are not recognizable by BLAST search and their homology to eukaryotic and bacterial ATP synthase subunits *a* and *b*, respectively, was recognized previously by detailed sequence analysis and hydropathic profiles [36],[37].

c dash indicates that no homologous proteins were identified in the *T. brucei* genome using BLAST search.

Comparison of bacterial, yeast and mammalian ATP synthase subunits to the *T. brucei* genome revealed *T. brucei* homologs of F_1_ subunits α, β, γ, δ, ε and F_0_ subunits *c* and OSCP. None of the other subunits found in other eukaryotic organisms have been identified. These are either absent from *Trypanosoma* mitochondrial ATP synthase, have been replaced by other proteins, or are so highly divergent that their relationship cannot be readily identified via sequence homology.

This observation prompted us to investigate the composition of the trypanosomal F_0_F_1_-ATP synthase. We identified 22 subunits in purified complexes of which 14 are unique to *T. brucei*. RNAi silencing of the subunit α and two novel components revealed these to be essential for PF cell viability and important for F_0_F_1_-ATP synthase structural integrity. We found a dramatic decrease in ATP production by oxidative phosphorylation in these silenced cell lines but no severe effect on substrate phosphorylation. The PF cells are sensitive to the ATP synthase inhibitor oligomycin regardless of the presence or absence of glucose in the medium. This is consistent with the RNAi data but contrary to earlier reports [15],[16]. Hence, regulation of mitochondrial energy generation in these parasitic organisms is more complex than previously thought.

## Materials and Methods

### Genes used in this study

Tb10.70.7760 (TAP_Tb7760, RNAi_Tb7760); Tb927.5.2930 (TAP_Tb2930, RNAi_Tb2930); Tb927.5.1710 (TAP_subunit *b*); Tb927.3.1380 (TAP_subunit β); Tb927.7.7430/Tb927.7.7420 (RNAi_subunit α).

### Plasmid construction

To create the vectors for inducible expression of C-terminally TAP-tagged proteins the ORFs were PCR amplified from *T. brucei* strain 427 genomic DNA using the following oligonucleotides:

TAP_Tb7760 Fwd – ACAAAGCTTATGCAGGGCAGTTGG

 Rev – ACAGGATCCAGCTGTGTGTCGGCC

TAP_sub *b* Fwd – CACAAGCTTATGATGCGCCGTG

 Rev – CACGGATCCCTCTACCTTTACATC

TAP_Tb2930 Fwd – ACAAAGCTTATGCGCCGTGTATC

 Rev – ACAGGATCCGTGATGGGCC

TAP_sub β Fwd – ACAAAGCTTATGCTGACTCGTTTCC

 Rev – ACAGGATCCGCTACTGGCTTG

The PCR products were cloned into pGEM-T easy vector (Promega), digested with BamHI and HindIII enzymes and ligated into the pLew79-MHT vector which contains *c*-myc, His, calmodulin binding peptide and protein A tags in that order [17],[18]. The last two tags are separated by a TEV protease cleavage site.

To create the construct for RNAi of Tb10.70.7760 and Tb927.5.2930 transcripts, fragments of 830 bp and 646 bp, respectively, were amplified by PCR using the oligonucleotides below and cloned into pZJM plasmid [19] via XhoI and HindIII restriction sites.

RNAi_Tb7760 Fw – CACAAGCTTGAAGCTCAGGACC

 Rev – CACCTCGAGGCAGAAACGCATC

RNAi_Tb2930 Fw – ACAAAGCTTATGCGCCGTGTATC

 Rev – CACCTCGAGTTCGGCCCGATC

The inducible RNAi plasmid for silencing ATP synthase subunit α was generated using the pQuadra system [20] as described in [4].

### Cell culture and generation of cell lines

*T. brucei* PF cells strains 29.13, transgenic for T7 RNA polymerase and the tetracycline (tet) repressor, were grown *in vitro* at 27°C in SDM-79 media containing hemin (7.5 mg/ml) and 10% FBS. The TAP-plasmids and RNAi plasmids were linearized with NotI enzyme and transfected into the cell line as described previously [21]. Synthesis of dsRNAi was induced by the addition of tet at 1 µg/ml concentration. The cells were counted using the Z2 Cell Counter (Beckman Coulter Inc.) and growth curves were generated for clonal cell lines over a period of 13 days. In TAP-tagged cell lines the expression of tagged protein was induced by 100 ng/ml of tet.

### SDS PAGE and Western blot analysis

The protein samples were fractionated by SDS-PAGE, blotted onto PVDF membrane and probed with monoclonal antibodies (mAb) anti-His_6_ (1∶2000, Invitrogen), anti-Rieske protein (1∶1000) (kindly provided by L. Simpson) and anti-alternative oxidase TAO (1∶25) (kindly provided by M. Chaudhuri), and polyclonal antibodies against trCOIV (1∶1000) [22], the F_1_ moiety of *Crithidia fasciculata* (kindly provided by R. Benne), which cross-reacts with the β subunit of the *T. brucei* complex (1∶1000) and against subunit *b* of *Leishmania tarentolae* (1∶2000) (kindly provided by L. Simpson), and developed using the ECL system (Roche).

### Immunoprecipitation of F_0_F_1_-ATP synthase complex

The mitochondrial vesicles were isolated from PF 1.7a cells as described previously [23] by hypotonic lysis followed by density gradient floatation in a 20–35% linear Percoll gradient. The enriched vesicles were lysed with 1% Triton X-100 and the lysate was clarified by centrifugation. The cleared supernatant was fractionated on a 10–30% glycerol gradient at 38,000 rpm for 5 hours (SW40 rotor, Beckman Instrument). The fractions were collected from the top. Immunoprecipitation of F_0_F_1_-ATP synthase complex using mAb64 was performed on pooled gradient fractions from the 10S region (fraction 3–5) and from the 40S region (fractions 17–21) using anti-mouse IgG-coated magnetic beads (Dynabeads M-450) as described previously [23]. The pulled down proteins were identified by LC-MS/MS analysis.

### Immunofluorescence assay (IFA)

Subcellular localization of the expressed tagged proteins within the cell was determined by IFA using polyclonal anti-*myc* (Invitrogen) as described [24]. Co-localization analysis was performed using mAb78 against mt heat shock protein 70 [23] coupled with Texas® Red-X conjugated secondary antibody (Invitrogen).

### Tandem affinity purification (TAP) of tagged complexes

The TAP protocol was adapted from the published method [18],[25],[26]. We purified the tagged complexes from 1–4×10^10^ cells by two complementary methods. Briefly, in method 1 the harvested cells were lysed by 1% Triton-X 100 and the tagged-complexes were isolated by IgG affinity chromatography. The bound complexes were eluted by TEV protease cleavage and fractionated on a 10–30% glycerol gradient by centrifugation for 5 h at 38,000 rpm at 4°C in an SW-40 Sorvall rotor [27]. The sedimentation profiles of the tagged complexes were monitored by Western blot analyses using anti-His_6_ mAb. Peak reactive fractions were pooled and further purified by Calmodulin affinity chromatography. In method 2 the tagged complexes were purified from cells lysed with 0.25% NP-40, cleared by low speed centrifugation and the supernatant was further lysed with 1.25% NP-40 and cleared by high speed centrifugation (40,000 rpm at 4°C in a Sorvall SW-55 rotor for 40 min). The tagged complexes were isolated by sequential binding to IgG and calmodulin affinity columns. This method was adapted from the published protocol [28].

### Mass spectrometry analysis

We prepared and analyzed the samples by gel-based and gel-free approaches as described previously [23],[24]. Peptides were identified using a Thermo Electron LTQ Linear Ion Trap Mass Spectrometer. The CID spectra were compared to the *T. brucei* protein database downloaded from GeneDB using TurboSequest software, and protein matches determined using PeptideProphet and ProteinProphet software [29],[30]. Proteins identified by at least two unique peptides with a minimum identification probability of 0.97 and in at least three TAP tag purified complexes are considered as putative subunits of ATP synthase complex. All of the proteins reported in this study were identified by multiple peptide matches except for two ATP synthase subunits, subunit ε (predicted M_w_ 8.6 kDa) and subunit *c* (predicted M_w_ 12.3 kDa), which were identified by only 1 peptide match.

### Digitonin fractionation, ATPase assay and ATP production assay

Crude mt preparations from the RNAi knock-down cell lines were obtained by digitonin extraction [31]. ATPase activity was measured based on release of free phosphate [32] as described [4]. Briefly, the reaction was started by addition of ATP to a final concentration of 5 mM; and where indicated, oligomycin and/or sodium azide was added to 2.5 µg/ml and 1 mM, respectively. After 20 min, 1.8 µl of 60% perchloric acid was added to 95 µl aliquots, the samples were kept on ice for 30 min, spun down and 90 µl of the supernatant was added to 0.5 ml of Sumner reagent [32], and absorbance was measured at 610 nm.

ATP production was measured as described [33]. Briefly, production of ATP was induced by 5 mM indicated substrates (succinate, pyruvate, α-ketoglutarate) and 67 µM ADP was added. Where indicated, 6.7 mM malonate or 33 µg/ml atractyloside were pre-incubated with mitochondria on ice for 10 min. The concentration of ATP was determined by a luminometer using the ATP Bioluminescence assay kit CLS II (Roche Applied Science).

### Blue-native polyacrylamide gel electrophoresis (BN-PAGE) and histochemical staining

The mt vesicles from 5×10^8^ cells were isolated by hypotonic cell lysis as described elsewhere [34] and lysed with 1% dodecyl maltoside; 50 µg and 100 µg of mitochondrial lysate and 75 µg of native high molecular weight marker (Amersham) was loaded per lane and analysed on a 3–12% gradient BN-PAGE gel. Immediately after the run, the gel was transferred into ATPase reaction buffer (35 mM Tris-HCl (pH 8.0); 270 mM glycine; 19 mM MgSO_4_; 0.3% [w/v] Pb(NO_3_)_2_; 11 mM ATP) for overnight incubation by slow agitation. The ATPase activity appears as a white precipitate. The gel was subsequently fixed in 30% methanol.

## Results

### Genome analysis

Mitochondrial F_0_F_1_-ATP synthase consists of up to 19 different subunits in yeast, and mammals [10],[35]. We searched the *T. brucei* genome database using these known subunits of the mitochondrial F_0_F_1_-ATP synthase and identified 7 homologs with varying degree of conservation as outlined in Table 1. The mitochondrial F_1_ subcomplex with its central stalk contains five different subunits designated α, β, γ, δ, ε and they are conserved among eukaryotes. We found that all five are also conserved in *T. brucei*, three of which were already annotated and two were mis-designated in GeneDB database. They are ATP synthase subunits α (encoded by two identical open reading frames Tb927.7.7420/Tb927.7.7430), β (Tb927.3.1380), and γ (Tb10.100.0070). Subunit δ (Tb927.6.4990) is currently annotated as subunit ε in GeneDB (consistent with the bacterial nomenclature) (Table 1). Since the GeneDB annotation for ATP synthase subunits otherwise follows the mitochondrial nomenclature we propose re-annotating it accordingly. The hypothetical protein encoded by (Tb10.70.2155) has similarity to the mitochondrial ATP synthase subunit ε and thus we propose re-annotating it as such (Figure S1).

While genes for all five F_1_ protein homologs were identified, only two of the 11 proteins that occur in the mitochondrial F_0_ subcomplex with its peripheral stalk were identified in the *T. brucei* genome. These are subunit OSCP (Tb10.6k15.2510) and subunit *c*, which is encoded by three distinct open reading frames Tb10.70.6340, Tb11.02.2950, Tb927.7.1470 that differ only at the N-terminus of the protein sequence (Figure S1). The finding of three genes that specify proteins related to subunit c is intriguing given the respiratory changes that occur during the life cycle of *T. brucei*. Subunit *a* (which is mitochondrially encoded) and subunit *b* (Tb927.5.1710) are not recognizable by BLAST search and their homology to ATP synthase subunits was recognized previously by detailed sequence analysis and hydropathic profiles [36],[37]. However, it should be noted that because the homology of Tb927.5.1710 to subunit *b* of other species is very low and limited to the N-terminal region of the protein, this identification should be treated as tentative. The homologs of the other conserved F_0_ subunits or of species-specific ATP synthase complex proteins were not identified by these searches. Since this analysis provided little information with respect to the identity of any other ATP synthase complex proteins including diverged or species specific proteins, we decided to obtain a detailed picture of the protein composition of this complex in *T. brucei* after purification using a combination of immunoprecipitation, blue-native polyacrylamide gel electrophoresis (BN-PAGE) and tandem affinity purification (TAP).

### ATP synthase complex immunoprecipitates

**Figure 1:**
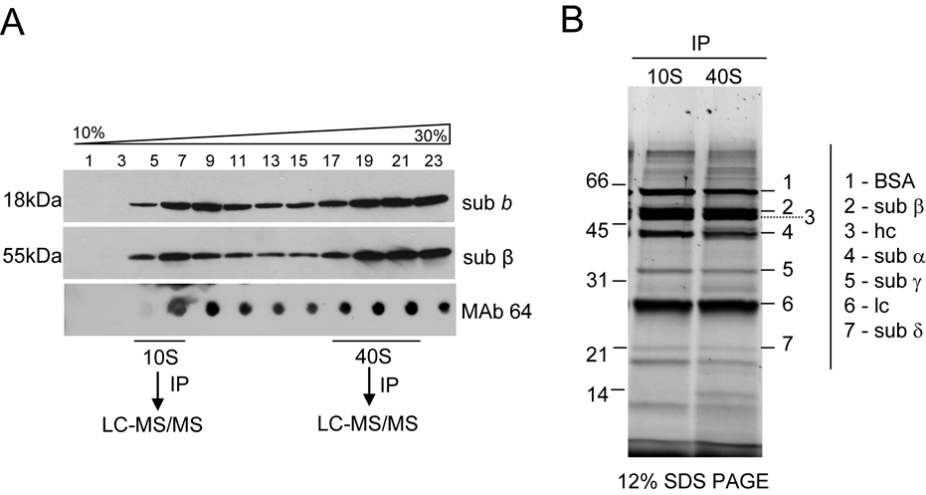
Glycerol gradient fractionation and immunoprecipitation of F_0_F_1_-ATP synthase complex from *T. brucei* mitochondria. (A) Western blot and native dot blot analyses of the 10–30% glycerol gradient-fractionated cleared mitochondrial lysate were performed using polyclonal antibodies against subunit *b* and β, and monoclonal antibody mAb64 to determine the sedimentation pattern of the F_0_F_1_-ATP synthase complex. MAb64 was further used to immunoprecipitate (IP) complexes from the 10S and 40S peaks and their protein compositions were analyzed by liquid-chromatography tandem mass spectrometry (LC-MS/MS). (B) Immunoprecipitated 10S and 40S complexes were fractionated on a 12% SDS PAGE gel and stained by Sypro Ruby. Protein bands corresponding to immunoglobulin heavy (hc) and light (lc) chains as well as predicted positions of F_1_ subunits α, β, γ and δ and the size standards are indicated.

Analyses of Triton X-100 lysate of highly purified mitochondria that was fractionated in glycerol gradients using antibodies specific for ATPase subunits identified two predominant peaks at ∼10S and ∼40S (Figure 1). Polyclonal antibodies that are specific for subunits β and *b* and monoclonal antibody mAb64 which recognizes a conformational epitope of an unidentified subunit of the F_1_ subcomplex (A. Panigrahi, unpublished) showed a similar sedimentation profile for two subunits in Western (upper panels) and native dot blot (lower panel) analyses. MAb64 was used to immunoprecipitate complexes from these two peaks and their protein compositions were analyzed by liquid-chromatography tandem mass spectrometry (LC-MS/MS). The immunoprecipitation of the pooled peak reactive fractions (10S and 40S) from the glycerol gradients was carried out in buffer containing 200 mM salt to increase the stringency. MS analysis of the immunoprecipitate from the 10S fraction revealed peptides from subunits α and β of the catalytic headpiece and subunits γ and δ of the central stalk. These subunits are part of the matrix-facing F_1_-moiety, which is not directly membrane bound. MS analysis of the 40S complex identified, in addition to these F_1_ subunits, OSCP protein and 9 hypothetical proteins of which homologs of three (Tb11.47.0022, Tb927.3.1690, Tb10.6k15.0480) had previously been identified as ATP synthase subunits in *C. fasciculata* [36] (Table 2). In agreement with MS data, a Sypro Ruby-stained gel of immunoprecipitated complexes revealed the presence of F_1_ subunits α, β, γ, and δ in the 10S immunoprecipitate, which predicted positions are designated and additional protein bands in the 40S immunoprecipitate (Figure 1B). Peptides predicted from the genes for subunits ε, *a*, *b* and *c* that had been found in *T. brucei* genome were not identified in this analysis although three of these proteins were detected by other procedures as shown below. The failure to detect expected subunits in the immunoprecipitates may be due to purification of incomplete ATP synthase complexes or due to the high hydrophobicity of these subunits and the presence of few potential tryptic cleavage sites.

**Table 2:** *Trypanosoma brucei* F_0_F_1_-ATP synthase subunits.

	Subunit	Protein ID^a^	Mw^b^	IP^c^		TAPtags^c^			BN gel^c^		
				10S	40S	Bands	M1	M2	F1	monomer	dimer
F_1_	alpha	Tb927.7.7420/7430*	63.5	√	√	√	√	√	√	√	√
	beta	Tb927.3.1380* ^,^ TAP	55.7	√	√	√	√	√	√	√	√
	gamma	Tb10.100.0070*	34.3	√	√	√	√	√	√	√	√
	delta	Tb927.6.4990*	20.1	√	√	√	√	√	√	√	√
	epsilon	Tb10.70.2155	8.6	-	-	-	√	√	-	-	-
F_0_	*a*	AAA97428	28	-	-	-	-	-	-	-	-
	*b*	Tb927.5.1710* ^,^ TAP	21.2	-	-	√	√	√	-	√	√
	*c*	Tb10.70.6340	12.3	-	-	-	-	√	-	-	-
	OSCP	Tb10.6k15.2510*	28.8	-	√	√	√	√	-	√	√
Associated proteins		Tb10.70.7760TAP	46.7	-	√	√	√	√	-	√	√
		Tb927.5.2930TAP	43.3	-	√	√	√	√	-	√	√
		Tb11.02.4120	27.5	-	√	√	√	√	-	√	√
		Tb10.6k15.0480*	17.1	-	√	√	√	√	-	√	√
		Tb927.3.1690*	17.1	-	√	√	√	√	-	√	√
		Tb11.47.0022*	20.2	-	√	√	√	√	-	√	√
		Tb927.7.840	14.5	-	√	√	√	√	-	√	√
		Tb11.03.0475	12	-	-	√	√	√	-	√	√
		Tb927.2.3610	16	-	√	√	√	√	-	-	√
		Tb927.3.2880	12.6	-	√	-	√	√	-	√	√
		Tb927.3.2180	17.9	-	-	-	√	√	-	√	√
		Tb927.5.3090	11.6	-	-	-	√	√	-	-	√
		Tb927.4.3450	13.7	-	-	-	-	√	-	√	√
		Tb927.8.3320	53.4	-	-	-	-	√	-	√	-

a GeneDB accession number except for subunit *a* (NCBI accession number).

b number indicates predicted molecular weight of the protein in kDa.

c √ indicates that the protein was identified by LC-MS/MS analyses, - not detected.

IP – immunoprecipitate of 10S and 40S complexes.

TAP – tandem affinity purification.

Bands – proteins were identified by gel band analysis of Sypro Ruby stained SDS PAGE gel.

M1, M2 – ATP synthase complex was purified by Method 1 or Method 2 (see Materials and Methods).

BN – proteins were identified in BN PAGE bands active for ATPase activity.

*: proteins identified in *Crithidia fasciculata* F_0_F_1_-ATP synthase complex [36].

TAP proteins used as baits.

### Tagged ATP synthase complexes and subunit composition

To obtain more detailed information about the composition of the ATP synthase complex and to assess the validity of the association of the novel proteins identified in the mAb64 immunoprecipitates we purified the F_0_F_1_-ATP synthase from *T. brucei* PF cells by tandem affinity purification (TAP) and analyzed the protein composition by LC-MS/MS analysis using published protocols [23],[24]. F_1_ subunit β (Tb927.3.1380) and F_0_ subunit *b* (Tb927.5.1710) as well as two randomly selected hypothetical proteins identified in the 40S complex (Tb10.70.7760 and Tb927.5.2930) were tagged resulting in cell lines TAP_sub β, TAP_sub *b*, TAP_Tb7760 and TAP_Tb2930.

**Figure 2:**
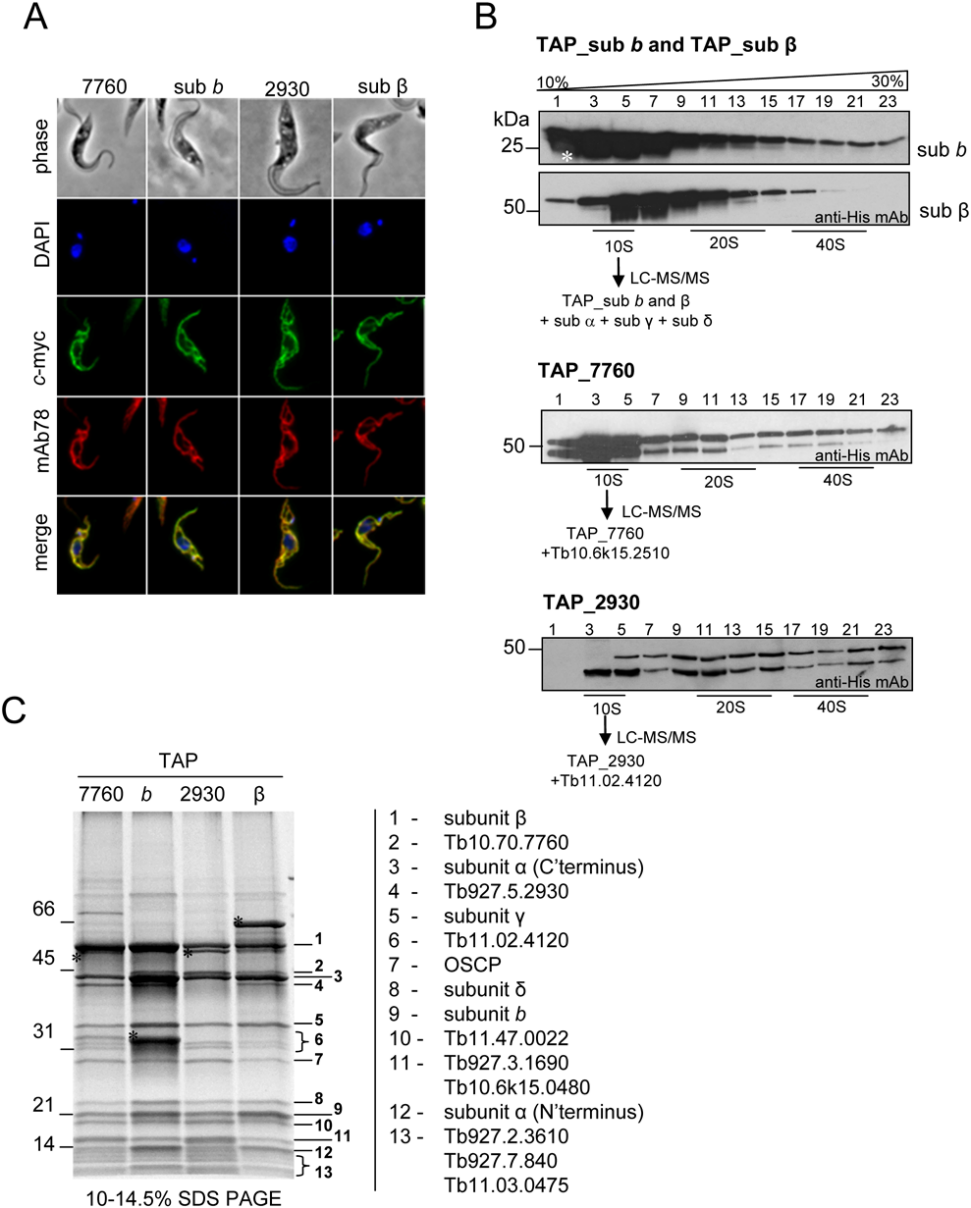
Subcellular localization of tagged subunits and tandem-affinity purification (TAP) of F_0_F_1_-ATP synthase complex from *T. brucei*. (A) Tagged subunits *b*, β, Tb7760 and Tb2930 of the F_0_F_1_-ATP synthase complex were visualized by fluorescence microscopy using polyclonal anti-*c* myc antiserum coupled with FITC-conjugated secondary antibody. Co-localization immunofluorescence was performed with monoclonal antibody mAb78 against the mt heat shock protein 70 [23]. Top row, phase-contrast light microscopy of *T. brucei* cells; second row, 4,6-diamidino-2-phenylindole (*DAPI*)-staining of nuclear and kinetoplast DNA; third row, localization of tagged proteins; fourth row, staining of mitochondrial hsp70; bottom row, merged fluorescence images. (B) Fractionation of TAP_sub *b*, TAP_sub β, TAP_Tb2930 and TAP_Tb7760 TEV eluates on 10–30% glycerol gradients. Fractions were collected from the top of the gradients. Aliquots of odd-numbered fractions were analyzed by SDS-PAGE and probed with anti-His_6_ mAb. Positive fractions designated by a black line were further subjected to the second affinity purification step. Proteins identified in 10S complexes by LC-MS/MS are shown. The asterisk in the TAP_sub *b* panel indicates the position of the 25-kDa His-tagged TEV protease. The sizes of the protein marker are indicated on the left. (C) TAP_Tb7760, TAP_sub *b*, TAP_Tb2930 and TAP_sub β complexes were purified by Method 2, separated on a 10–14.5% polyacrylamide Tris-glycine gel and stained with Sypro Ruby. Numbers on the right indicate the results of MS analysis of individual bands. The asterisks indicate the tagged proteins. The sizes of the protein marker are indicated.

Immunofluoresence assays using anti-*myc* mAb (anti-tag) showed that TAP_sub β, TAP_sub *b*, TAP_Tb7760 and TAP_Tb2930 localized to the mitochondrion (Figure 2A). These results confirm the mitochondrial localization of these proteins and indicate that the TAP tags did not interfere with mitochondrial import and providing validity for purifying the F_0_F_1_-ATP complex.

Western analysis of the tagged complexes purified from cell lysate by IgG affinity chromatography, released by TEV cleavage, and fractionated on 10–30% glycerol gradients revealed sedimentation profiles that differed from those of untagged complexes from mitochondrial lysate (Figure 2B, compare to Figure 1). All tagged subunits were detected throughout the gradients, with the majority of tagged subunits β, *b* and 7760 sedimenting at around 10S. This may reflect the effect of over-expression of the tagged protein as well as potential effects of the tag on the assembly, stability, composition, and/or compactness of the complexes. The bands that are smaller than the predicted full length proteins and that are especially evident in glycerol gradient fractions of TAP_Tb7760 and TAP_Tb2930 are most likely degradation products of the tagged proteins reflecting the abundance of endogenous proteases in the whole cell lysates or results of a proteolytic degradation due to protein overproduction.

The tagged complexes were further purified from pooled gradient fractions corresponding to ∼10S (fractions 3–5), ∼20S (fractions 7–13) and ∼40S (fractions 17–21), as indicated by the underlines in Figure 2B, using the second affinity purification step and analyzed by mass spectrometry. LC-MS/MS analyzes of the complexes from the 20S and 40S fractions from the four TAP-tag purifications identified 18 proteins that were all present in all 40S fractions and almost all of the 20S fractions (Table S1, Method 1). This very similar composition suggests that the 40S complex may be a dimer or oligomer of the 20S complex which may represent a F_0_F_1_-ATP synthase monomer. ATP synthase subunit α, β, γ, ε and *b* were identified in ∼10S complexes from cells in which subunits β or *b* were tagged (i.e. TAP_sub β, TAP_sub *b*) perhaps representing the catalytic headpiece attached to the central stalk with bound subunit *b* (Figure 2B). However, only the OSCP protein (Tb10.6k15.2510) and hypothetical protein Tb11.02.4120 were identified in addition to the bait protein in ∼10S complexes from TAP_Tb7760 and TAP_Tb2930 cells respectively (Figure 2B). Perhaps, these represent small sub-complexes of the tagged protein and binding partner(s) that are a consequence of partial assembly or TAP-tag induced disruption of the ATP synthase complex. Subunit ε, which is a small protein with a predicted mature size of 6.7 kDa was identified only in the ∼10S complexes from TAP_sub β cells. This may indicate a weaker association with the central stalk of the ATP synthase (Table S1).

To investigate the sedimentation characteristics of the tagged complexes, glycerol gradient fractions of TAP_sub β and TAP_sub *b* were subjected to SDS PAGE followed by Sypro Ruby staining (Figure S2). The staining revealed that the majority of the tagged proteins migrated at S values <10. At greater S values there was a higher relative abundance of subunits α and β compared to approximately equal amounts of the other subunits, which is consistent if there are three copies of subunits α and β per complex as seen in other organisms.

We also purified the F_0_F_1_-ATP synthase complex by a complementary affinity purification method that entails two steps of NP-40 treatment and is designed to purify intact membrane complexes ([28], see method 2 in Methods section). SDS-PAGE analysis of all four tagged complexes showed very similar protein profiles, which differed mainly in the position of tagged bait (Figure 2C). Interestingly, in case of TAP_2930 and TAP_7760 the band corresponding to non-tagged endogenous protein was not apparent in the purified complexes, whereas in the case of subunits β and *b* such a band could be detected. This may imply that there is one copy of Tb7760 and Tb2930 protein per complex, while there are three copies of subunit β and potentially two or more copies of subunit *b* per complex. All visible gel bands were individually analyzed by mass spectrometry and the respective proteins were identified (Figure 2C, Table 2). The 15 proteins that correspond to F_1_ subunits α, β, γ and δ, F_0_ subunits *b* and OSCP, and nine hypothetical proteins (Tb10.70.7760, Tb927.5.2930, Tb11.02.4120, Tb11.47.0022, Tb927.3.1690, Tb10.6k15.0480, Tb927.2.3610, Tb927.7.840, Tb11.03.0475) were identified. In addition, one peptide for F_0_ subunit *c* was identified only in complexes from TAP_Tb2930 cells (Table S1). Its small size (mature size of 8 kDa) and high hydrophobicity make the identification of its peptides by routine mass spectrometry analysis challenging. Subunit α was detected as separate ∼14 kDa and ∼44 kDa N- and C- terminal fragments, respectively (Figure S3). This implies a specific posttranslational cleavage that has been previously reported for *L. tarentolae* [38] and suggests that this cleavage occurs *in vivo* and it is not an artifact of purification. Six other proteins were identified in three samples when the SDS-PAGE step was omitted and samples were directly submitted to trypsin cleavage and LC-MS/MS analysis. These proteins are most likely associated with the F_0_F_1_-ATP synthase although their relative concentration (stoichiometry) may be lower compare to the 15 proteins identified by gel-band analysis (Table 2, Table S1). The mitochondrial encoded subunit *a* was not identified, perhaps due to transient association with subunit *c* and/or its high hydrophobicity, membrane association, non-migration into SDS-PAGE gels and few potential trypsin cleavage sites.

Fourteen novel proteins found associated with the ATP synthase complex are currently annotated as hypothetical proteins in the GeneDB database. Direct comparison to the known subunits of yeast and mammalian ATP synthases did not reveal any similiarity. To explore the homology of these proteins to any other proteins we performed PSI-BLAST searches against the “nr” NCBI database and CDD, PFAM, PROSITE, and InterPro domain searches. We found that these proteins appear to be unique to the order *Kinetoplastida* and that they share no apparent motifs and similarities with any proteins outside of these organisms.

In summary, we find the trypanosomal F_0_F_1_-ATP synthase complex is composed of up to 22 subunits. This complexity is similar to that seen in higher eukaryotes, although the degree of subunit sequence similarity to those in higher eukaroytes is very low or none.

### Native *T. brucei* F_0_F_1_-ATP synthase

**Figure 3:**
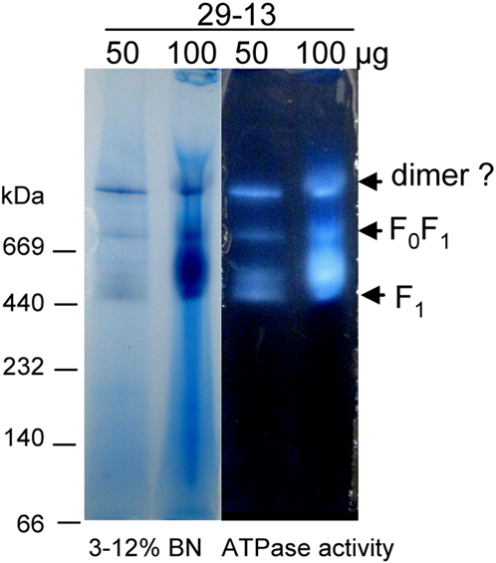
Analysis of the supramolecular organization of F_0_F_1_-ATP synthase complex from PF *T. brucei*. Mt membranes from parental 29-13 cell line were solubilized by dodecyl maltoside and the mt complexes were separated on 3–12% BN PAGE. ATP synthase F_1_ particles, F_0_F_1_ monomer and putative dimer were identified by lead phosphate precipitates formed during the in-gel ATP hydrolysis assay followed by LC-MS/MS analysis of the corresponding gel bands. The sizes of the native high molecular weight marker (Amersham) are indicated.

The *T. brucei* ATP synthase complex was examined using blue-native (BN) PAGE in which the charge shift induced by the binding of Coomassie Blue to proteins is used to separate and visualize membrane complexes under native conditions [39]. BN PAGE of dodecyl maltoside-solubilized mitochondria followed by ATPase activity staining revealed three predominant stained bands of which the lower band appears as a doublet, with apparent molecular weights of ∼450 kDa, ∼700 kDa, and >1 MDa, respectively, relative to the 440 kDa and 669 kDa marker proteins (Figure 3). The three bands active for ATPase activity were excised from the BN gels, destained, digested with trypsin and analysed by LC-MS/MS. We identified only the known subunits of the F_1_ moiety in the lower doublet, suggesting that it corresponds to the dissociated F_1_ moiety of ATP synthase. The upper two bands contained in addition to the the F_1_ moiety proteins the 16 other proteins which were identified in TAP-tagged purified complexes (Table 2, Table S1). This reinforces the likelihood that these proteins are novel subunits of F_0_F_1_-ATP synthase complex or are at least tightly associated with this complex. Two proteins (Tb927.2.3610 and Tb927.5.3090) were identified only in the >1 MDa gel band, implying specific association with F_0_F_1_dimers but additional studies are neccessary to assess this.

The molecular mass observed for the smallest band corresponds well to that estimated for the F_1_ moiety. This smaller band of the doublet might represents the F_1_ headpiece (subunit α and β) and the larger this headpiece together with central stalk (subunit αβγδε) with or without a ring of subunit *c* as has been shown for mammalian F_1_ [14]. The observed size of the middle BN gel band corresponds roughly to the ∼820 kDa sum of 22 proteins identified in purified complexes that may be stable constituents of this complex (Table 2) and thus may correspond to the F_0_F_1_ monomer. The upper band thus may represent a dimer or oligomer of the ATP synthase complex. This is with agreement with BN PAGE analysis of other trypanosomatid species, *L. tarentolae* and *Phytomonas serpens*, which also revealed dissociated F_1_ ATP synthase particles and monomeric and dimeric/oligomeric F_0_F_1_-ATP synthase complexes [22],[40].

### Depletion of subunit α, Tb2930, or Tb7760 inhibits growth of PF *T. brucei*

**Figure 4:**
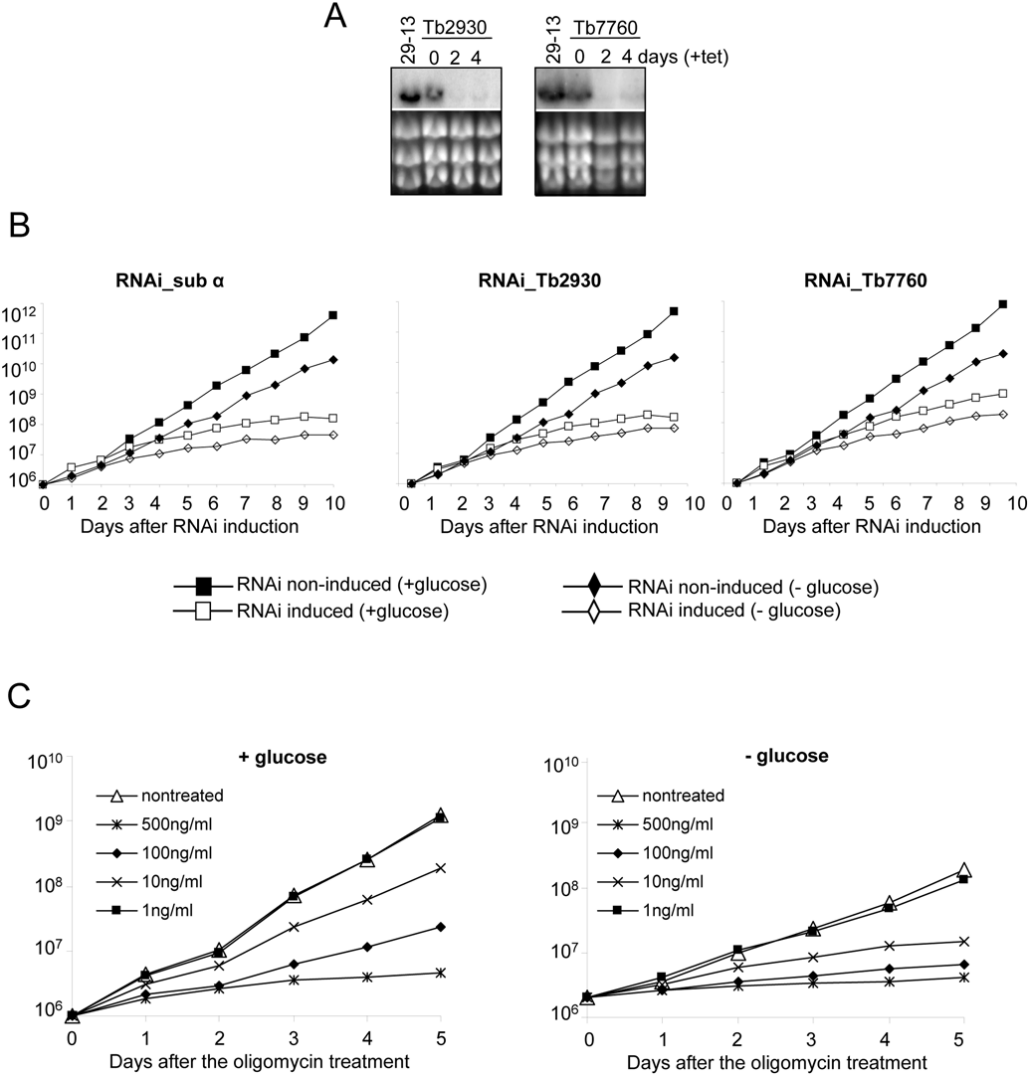
Subunits α, Tb7760 and Tb2930 are important for the *in vitro* growth of procyclic parasites. (A) Northern analyses of the corresponding mRNAs for Tb7760 and Tb2930 RNAi cell lines with the days sampled indicated; and stained gels of rRNAs in the lower panel serving as loading controls. (B) Growth curves of uninduced and induced RNAi-sub α (left), RNAi_Tb2930 (middle panel) and RNAi_Tb7760 (right) cell lines in the presence or absence of glucose. Cells were maintained in the exponential growth phase (between 10^6^ and 10^7^ cells/ml) and cumulative cell number represents the normalization of cell density by multiplication with the dilution factor. (C) Growth of the 29-13 procyclic cell line in the presence or absence of glucose and in response to treatment with the indicated concentrations of oligomycin. Cells were cultured and their growth measured as described for (B).

**Figure 5:**
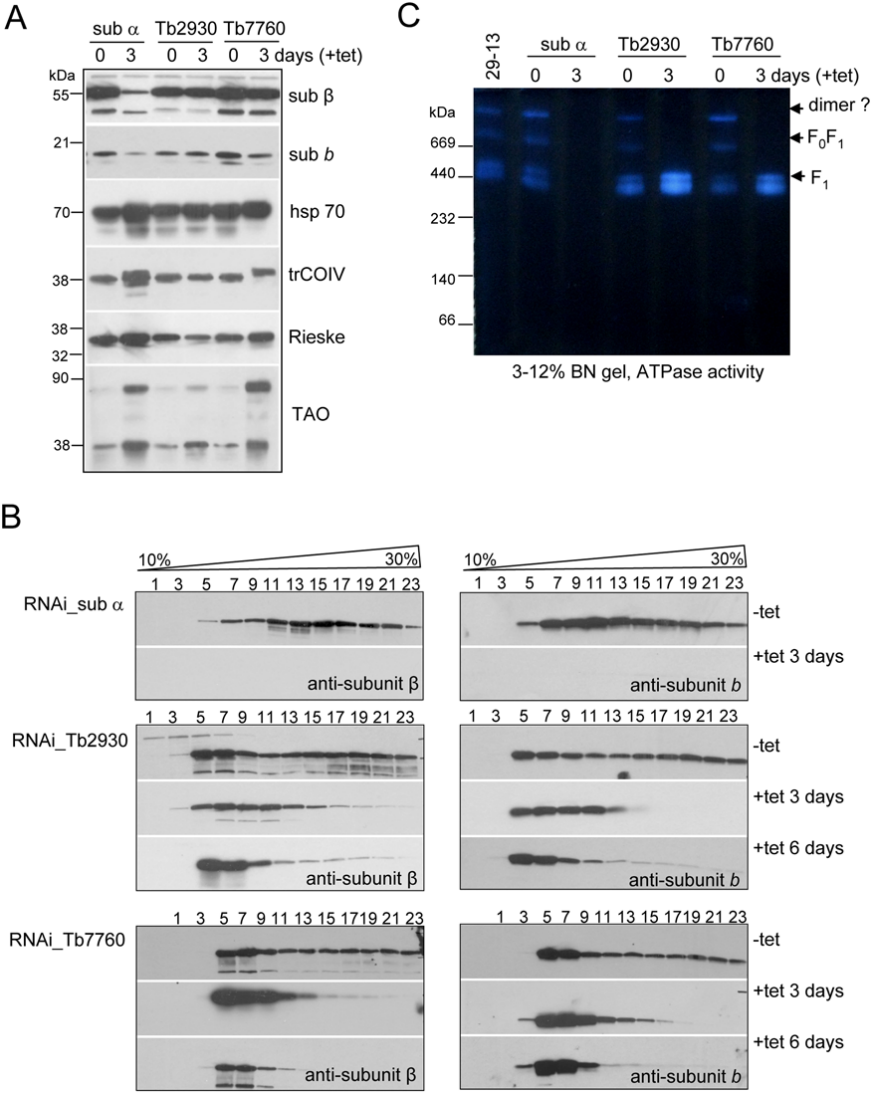
Effect of RNAi silencing of subunits α, Tb7760 or Tb2930 on steady-state abundance and integrity of F_0_F_1_-ATP synthase complex. (A) The steady state abundance of examined proteins was analyzed by Western analysis of lysed crude mitochondria (7.5 µg of proteins per well) prepared from RNAi-induced cells after 3 days and from uninduced control cells. The blots were probed with antibodies against subunits β, *b*, trCOIV, Rieske protein, mt alternative oxidase (TAO) and mt hsp 70 as a loading control. (B) Western analyses of glycerol gradient fractions (numbered from top to bottom) from crude mitochondrial lysate of RNAi cell lines grown in the absence (−tet) or presence (+tet) of tetracycline for 3 and 6 days are shown. (C) In-gel ATP hydrolysis activity of *T. brucei* F_0_F_1_-ATP synthase complexes after ablation of subunit α, Tb7760 or Tb2930. Mitochondrial preparations were solubilized using dodecyl maltoside and separated by 3–12% BN PAGE. In-gel ATP hydrolysis/lead phosphate precipitation assay revealed bands representing putative monomeric and dimeric ATP synthase complexes and free F_1_ particles. The sizes of the native high molecular weight marker (Amersham) are indicated.

To assess the requirement of the ATP synthase complex in PF stage of *T. brucei* and to evaluate the functional association of the newly identified subunits of the F_0_F_1_-ATP synthase complex we constructed cell lines in which the expression of subunits α, Tb927.5.2930 and Tb10.70.7760 can be silenced using RNA interference (RNAi). RNAi of the ATP synthase subunit α was mediated by a stem-loop construct containing a 530 bp fragment of the gene's coding region. RNAi of the Tb2930 and Tb7760 was mediated by pZJM construct. Both vectors allow tetracycline (tet)-dependent and thus regulatable expression of dsRNA which results in RNAi-mediated degradation of the target mRNA. The efficiency of RNAi was confirmed by Northern blot analysis showing that mRNA for Tb2930 and Tb7760 is almost eliminated by day 2 after RNAi induction (Figure 4A). Tb7760 and Tb2930 protein levels could not be directly assessed in the RNAi cells due to the lack of antibodies against these proteins. Since a specific antibody against F_1_ subunit β is available and the stability of subunit α and β is mutually dependent [4],[5] we confirmed the RNAi efficiency of RNAi_subα cell line by Western blot analysis (Figure 5A). After the addition of tet into the culture medium the growth of RNAi_subα, RNAi_Tb2930 and RNAi_Tb7760 cells lines was strongly inhibited and almost complete cessation of growth was evident by day 4 (Figure 4B).

These observed results are intriguing because it has been proposed that, under the growth conditions used in our RNAi studies (i.e. 6 mM glucose), the function of ATP synthase is not crucial for procyclic cell survival. This was concluded from the observations that in the presence of glucose these cells are 1000-times less sensitive to the ATP synthase inhibitor oligomycin than in the absence of glucose and that depletion of subunit β by RNAi led to cell death in glucose-depleted, but not in glucose-rich medium [15],[16]. This discrepancy between our observations and published data led us to examine the growth rate of RNAi induced cells and the oligomycin sensitivity of our cell lines in the absence of glucose in the media. The parental 29–13 cells (derived from strain 427) and RNAi non-induced cells were grown in the absence of glucose for three weeks. Interestingly, the doubling time for the cells grown in the presence of glucose was between 13.9–15.5 hours, whereas the cells grown in the absence of glucose grew much slower with the doubling time reaching the values of 19.7–21.4 hours (Table 3). RNAi induction of RNAi_subα, RNAi_Tb2930 and RNAi_Tb7760 cell lines increased the doubling time significantly to 34.5, 34.5 and 28.2 hours, respectively (Figure 4B, Table 3). Addition of 1 ng/ml oligomycin to the 29–13 cell grown in the presence or absence of glucose had no effect (Figure 4C, Table 3). Higher concentrations of oligomycin slowed growth significantly for both cell lines in a similar manner. At the highest concentration of oligomycin (1 µg/ml) both cell lines died. At intermediate concentration of oligomycin (500 ng/ml and 100 ng/ml), the cells cultivated in presence of glucose survived, although they were radically elongated, thin, and did not proliferate, whereas cells cultivated in the absence of glucose died. These results suggests that the 29–13 procyclic cells are more sensitive to oligomycin treatment than EATRO1125 cells used in the published studies [15],[16] and ATP synthase plays an important role in the growth and proliferation of these cells even in the presence of glucose.

**Table 3:** Effect of RNAi and oligomycin on the doubling time of procyclic *T. brucei* cell lines growing in the presence (+) or absence (−) of glucose in the media.

Cell line	Doubling time	
	+glucose	−glucose
RNAi subunit α – NON	15.5	21.4
RNAi subunit α – IND	34.5	38.0
RNAi Tb2930 – NON	15.3	21.3
RNAi Tb2930 – IND	34.5	36.0
RNAi Tb7760 – NON	14.5	20.8
RNAi Tb7760 – IND	28.2	32.6
29-13	13.9	19.7
29-13+1 ug/ml oligomycin	34.4	41.7
29-13+500 ng/ml oligomycin	34.9	41.4
29-13+100 ng/ml oligomycin	26.7	37.4
29-13+10 ng/ml oligomycin	17.6	30.4
29-13+1 ng/ml oligomycin	14.3	18.4

### Silencing of subunits α, Tb2930 or Tb7760 disrupts the ATP synthase complex

Western analysis of crude mitochondrial fractions prepared three days after RNAi induction of subunit α revealed a substantial loss of subunits β and *b* compared to non-induced cells (Figure 5A). The same Western analysis using the crude mitochondrial fraction from Tb2930 and Tb7760 RNAi-induced cells showed almost no changes in steady-state abundance of subunits β and *b*, suggesting that the F_1_ moiety together with subunit b was still assembled and stable in these cells (Figure 5A). To investigate potentially secondary effects on mt membrane biogenesis, we probed the same samples with antibodies against two subunits of the respiratory complexes III and IV. The abundance of the subunit trCOIV and Rieske protein remained unaltered in the analyzed cells. However, the level of the alternative oxidase protein was increased by day 3 in all examined RNAi cell lines suggesting that cells may have undergone some compensation for the perturbation of the classical respiratory pathway. Mt heat-shock protein (hsp) 70 was used as a loading control (Figure 5A).

To further characterize the consequences of a lack of subunit α, Tb2930 or Tb7760, all of which resulted in slower growth, we investigated the structural integrity of the ATP synthase complex. Crude mitochondrial lysates of RNAi cells, in which subunits α, Tb2930, or Tb7760 were expressed or repressed for 3 and 6 days, were fractionated on glycerol gradients and examined by Western analysis using the polyclonal antibodies against subunits β and *b*. In RNAi-sub α non-induced cells these two subunits co-sedimented in glycerol gradient fractions 5–23 (10–50S). However, no signal was observed with glycerol gradient fractions of crude mt lysate from 3 days RNAi-sub α induced cells (Figure 5B). This is in agreement with the reduced level of the steady state abundance of the subunit β and *b* (Figure 5A). These findings are similar to those observed in yeast [41], where the absence of the α subunit led to reduced levels of subunits β and b, presumably because the F_0_F_1_-ATP synthase does not assemble properly and its unincorporated subunits are degraded and/or mislocalized. In contrast, silencing of the Tb2930 and Tb7760 genes had a strong qualitative effect on integrity of the complex, resulting in a shift of complexes from higher to lower S values. This effect was even more pronounced at day 6 after RNAi induction when most of the signal for subunit β and *b* was found in fractions 5–7 (Figure 5B). Based on our data from immunoprecipitation experiments we may conclude that the F_0_F_1_ ATP synthase is disrupted in the absence of Tb2930 and Tb7760 and only the free F_1_ particles with bound subunit *b* are observed by Western blot. To assess whether sedimentation of other mt complexes does not change we used monoclonal antibody mAb52, which recognizes a native epitope of the NADH-ubiquinone oxidoreductase complex [23]. No significant changes were observed (Figure S4).

The influence of lack of subunit α, Tb7760, or Tb2930 on the ATP synthase assembly was further examined by BN PAGE analysis of dodecyl-maltoside-solubilized mitochondria in combination with activity-based staining, as described above (Figure 5C). For the parental 29.13 cells as well as for uninduced controls, the ATP synthase was found as monomer, putative dimer, and also as free F_1_ particles, as observed in Figure 3. For the subunit α-silenced cells, no ATPase activity was detected. This is in agreement with the experiments described above showing that F_1_ and F_0_F_1_ complexes are absent from the mitochondria of RNAi-sub α silenced cells. For subunit Tb2930- and Tb7760-silenced mitochondria, activity based bands representing the putative F_1_F_0_ monomer and dimer disappeared. In contrast, abundance and activity of the free F_1_ moiety both increased. Altogether, these analyses indicated that subunit Tb2930 and Tb7760 are required for a correct assembly of the other ATP synthase subunits and/or for the stability of the supramolecular structure of the complex.

### Subunits α, Tb2930, and Tb7760 are essential for ATP synthesis but only subunit α is also essential for ATP hydrolysis

**Figure 6:**
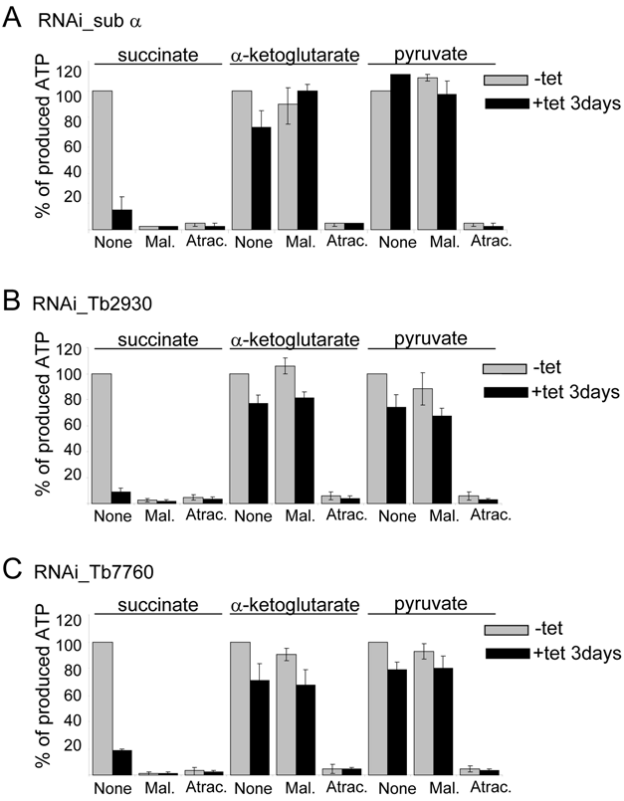
Effect of RNAi-mediated ablation of subunits α, Tb7760 or Tb2930 on mitochondrial ATP production. Crude mitochondrial preparations from uninduced and 3-day RNAi-silenced RNA_sub α (A), RNAi_Tb2930 (B) and RNAi_Tb7760 (C) cells were obtained by digitonin extraction and ATP production in the three mitochondrial pathways was measured individually. ATP-production was triggered by the addition of ADP plus one of the following substrates, succinate, α-ketoglutarate and pyruvate/succinate. Uninduced cells (−tet) are shown in grey, induced (+tet) are shown in black. The tested substrate is indicated at the top. Malonate (Mal.), a specific inhibitor of succinate dehydrogenase, was used to inhibit ATP production by oxidative phosphorylation and atractyloside (Atrac.) was used to inhibit import of ADP into mitochondria. Addition of these compounds to the sample is indicated at the bottom of each panel. ATP production in mitochondria isolated from uninduced cells and tested without additions of malonate or atractyloside (None) was set to 100%. The bars represent means expressed as percentages from three independent RNAi inductions. Standard deviations are indicated.

Mitochondrial ATP is produced in PFs via three different pathways that can be assayed in isolated intact mitochondria [33]. (i) Succinate appears to be the main substrate for oxidative phosphorylation with succinate dehydrogenase loading the respiratory chain with electrons, which generates a proton gradient that drives mitochondrial F_0_F_1_ ATP synthase. (ii) α-ketoglutarate induces ATP production by substrate level phosphorylation occurring in a partial citric acid cycle and (iii) pyruvate induces ATP production by substrate level phosphorylation occurring in the acetate-succinate CoA transferase/succinyl-CoA synthetase cycle. In all three knockdown cell lines the succinate dehydrogenase-dependent ATP production was reduced 80–90% at day three after RNAi induction (Figure 6A, B, C). Both pyruvate and α-ketoglutarate-induced ATP synthesis appear somewhat decreased upon down-regulation of Tb2930 and Tb7760 proteins (Figure 6B, C); suggesting that other mitochondrial processes may be secondarily affected by the silencing of these two subunits. Taken together, these results showed that subunits α, Tb2930 and Tb7760 are critical for ATP synthesis by oxidative phoshorylation in PF stage *T. brucei*.

In addition to ATP synthetic activity the ATP synthase complex possesses also ATP hydrolytic activity, but this reverse function is not dependent on the F_0_ moiety. ATPase activity was measured via release of free phosphate in digitonin-extracted mitochondria prepared from non-induced and from RNAi-induced cells. In non-induced cells the concentration of free phosphate was 80±3.7, 96±3.1 and 95±8.4 nmol in RNAi_α, RNAi_2930 and RNAi_7760 samples, respectively. This minor increase of ATPase activity observed in non-induced RNAi-Tb7760 and Tb2930 compared to non-induced RNAi-sub α cell line may be caused by leaky transcription of dsRNAi even in the absence of tet, thus inducing a partial RNAi phenotype. This effect was also noticeable in Western analysis of glycerol gradient fractions, where an accumulation of 10S complexes is obvious in non-induced RNAi-Tb7760 and Tb2930 cells (Figure 5B, middle and lower panels). Leaky RNAi is not unusual in this system [19],[42].

Oligomycin is a specific inhibitor of the F_0_F_1_ ATP synthase complex, putatively binding on the interface of subunit *a* and *c*-ring oligomer and blocking the rotary proton translocation in F_0_. If the enzyme is well-coupled, the activity of F_1_ is also blocked.

After the oligomycin treatment the concentration of free phosphate decreased down to 54±0.7 nmol, 59±8 and 61±0.7 in non-induced RNAi_α, RNAi_2930 and RNAi_7760 samples, respectively, which corresponds to reduction of ATPase activity by 33%, 37% and 36%.

**Figure 7:**
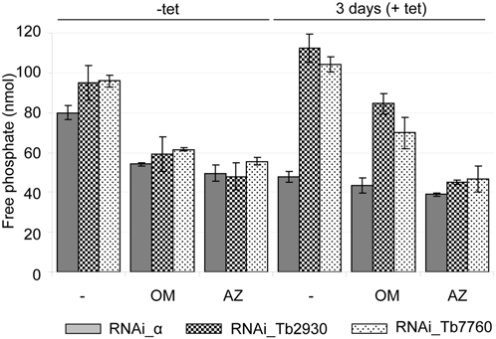
Effect of RNAi-mediated ablation of subunits α, Tb7760 or Tb2930 on mitochondrial ATPase activity. Crude mitochondrial preparations from uninduced and 3 days RNAi-silenced cells were obtained by digitonin extraction and ATPase activity was assayed by measuring release of free phosphate. Shown are data for RNAi_sub α (grey columns), RNAi_Tb2930 (small checker board columns) and RNAi_7760 (dotted diamonds columns) cell lines uninduced and induced for three days. ATP synthase inhibitors oligomycin (OM, 2.5 µg/ml) and azide (AZ, 1 mM) were added as indicated. Average numbers for three assays are shown, using extract preparation from three independent RNAi experiments.

Azide, an inhibitor of both F_0_F_1_ and the F_1_ moiety alone decreased the ATPase activity slightly more by 38%, 50% and 42% (Figure 7). This is comparable to oligomycin- and azide-induced inhibition observed in other experiments with crude mt fractions from *T. brucei*, indicating the presence of other ATP hydrolytic activities in these crude mt preparations [43],[44]. Silencing of sub α for three days led to a reduction of the concentration of free phosphate down to 48±3.8, which is comparable to the reduction obtained with oligomycin and/or azide. Addition of oligomycin and/or azide to these extracts resulted in minor further decreases of ATPase activity (Figure 7). These results demonstrate that the azide- and oligomycin- sensitive ATPase activities in these extracts were almost completely abolished, consistent with the results from the Western and BN PAGE analyses (Figure 5B, C). Thus, silencing of ATP synthase subunit α resulted in extensive and specific reduction of both azide- and oligomycin- sensitive activities of F_0_F_1_-ATP synthase complex.

Different results were observed for subunits Tb2930 and Tb7760. When the Tb2930 and Tb7760 subunits were silenced for three days, the total amount of the free phosphate increased to 112±5.2 and 104±7.6 nmol, respectively (Figure 7). This ATPase activity was still slightly oligomycin sensitive, suggesting that some of the 20S and 40S intact complexes remained in the mitochondria after three days of RNAi induction, consistent with the results from the Western analyses (Figure 5B). It should be noted that oligomycin in high concentrations also affects the activity of mitochondrial F_1_ and Na^+/^K^+^ ATPases [45],[46]. Treatment with azide decreased the amount of the free phosphate down to 45±6.3 and 46±6.8 nmol, respectively, which is comparable to the levels seen in non-induced oligomycin- or azide-treated cells (Figure 7). These results show that after the RNAi induction the azide-sensitive and oligomycin-insensitive ATPase activity increased significantly compared to non-induced cells. Thus, the loss of Tb2930 and Tb7760 subunits disrupts the F_0_F_1_ complex, resulting in release of functional F_1_ sector.

## Discussion

We report here the composition of the *T. brucei* F_0_F_1_-ATP synthase complex and its importance for the survival of the procyclic stage of the parasite. ATP synthase complexes and subcomplexes were purified by a combination of tandem-affinity chromatography, glycerol gradient sedimentation, immunoprecipitation, and blue native gel electrophoresis. The complexes were found by mass spectrometry to be composed of up to 22 subunits with molecular masses ranging from 8.6 to 55.7 kDa. Eight of these proteins are related to F_1_ subunits α, β, γ, δ, and ε and F_0_ subunits *b*, *c* and OSCP. The other 14 proteins have no recognizable eukaryotic counterparts and thus appear to be specific to *Trypanosoma*. The association of two of the novel proteins with the F_0_F_1_-ATP synthase complex was verified using reciprocal TAP tag analyzes, which also confirmed the association of other novel components. Some of these F_0_F_1_ associated novel proteins may have functionally and structurally replaced the accessory subunits *d*, *e*, *g*, *h*, IF1 that are conserved among the eukaryotic mitochondrial F_0_F_1_-ATP synthases. Gene silencing studies show that F_0_F_1_-ATP synthase subunit α and two of the novel proteins are essential for structural integrity of the F_0_F_1_-ATP synthase complex and for survival of PF *T. brucei*.

The 22 proteins identified in the F_0_F_1_-ATP synthase complex have an approximate total mass of ≤820 kDa. This size was calculated using the predicted protein mass without the predicted mt signal peptides, which are cleaved after the import into mitochondria and assuming that the monomer contains three subunits each of α and β and 10 subunits *c* (Table S1). This predicted molecular weight of the F_0_F_1_-ATP synthase complex roughly corresponds to that of an ATP hydrolytic complex that was resolved by BN PAGE, consistent with it being the F_0_F_1_-ATP synthase monomer. The BN PAGE also revealed bands consistent with dissociated F_1_-ATPase particles and a putative dimer of the complex. The *Trypanosoma* F_0_F_1_-ATP may exist as a dimer *in vivo*, which might be essential for biogenesis of cristae as has recently been shown for yeast and mammalian mitochondria [47],[48]. This profile is similar to those observed for mammalian and yeast F_0_F_1_-ATP synthase complexes on BN PAGE, although their molecular masses are only between 550–580 kDa [12], [49]–[51].

The mitochondrially encoded subunit *a* was not identified in our purified F_0_F_1_-ATP synthase complex preparations. This may due to its possible transient association with the oligomeric *c*-ring as has been seen in other systems [52] and indeed this subunit was identified in *Escherichia coli* only using cross-linkers [53]. Subunit ε and the small and hydrophobic subunit *c* were each identified in only one TAP-tag experiment. This probably reflects technical difficulties with routine mass spectrometric identification of the proteins which are smaller than 10 kDa, have few tryptic fragments, are strongly hydrophobic, and are membrane associated.

The RNAi knockdown studies showed that F_0_F_1_-ATP synthase is essential for growth of the PF *T. brucei* cells. Knockdown of subunit α expression resulted in dramatic loss of the complex while knockdowns of Tb2930 or Tb7760 protein expression resulted in dissociation of the complex and release of the F_1_ subcomplex, showing that these subunits are essential for complex integrity. ATP synthase monomer and dimer and their ATPase activities were not detected by BN PAGE analyses of mitochondria from induced RNAi-Tb7760 and -Tb2930 cells. However, dissociated F_1_ particles were present and had increased ATPase activity in the BN PAGE gel assay and in our metabolic assays. This resembles the result of deletion of yeast subunit *d* (ATP7), *f* (ATP17) or *h* (ATP14), which result in correct assembly of the F_1_ moiety, its dissociation from the F_0_ moiety, and increased turnover of unassembled F_0_ subunits [54]–[56]. Whether the novel subunits Tb7760 and Tb2930 have functional homologs in other systems remain to be determined. In contrast to subunit Tb7760- and Tb2930-depleted cells, purified mitochondria from RNAi_sub α induced cells lacked all three complexes active for ATPase activity, and oligomycin- and azide-sensitive ATPase activities were almost completely abolished. These results resemble those observed in yeast where the absence of the α subunit resulted in absence of the F_1_ moiety, impaired assembly of the F_0_ moiety, and degradation and/or mislocalization of its unincorporated subunits [41]. While we did not assess the status of the F_0_ moiety directly we conclude that the novel subunits Tb2930 and Tb7760 are required for structural integrity of the F_0_F_1_-ATP synthase while subunit α is essential for proper assembly of the F_1_ sector and the integrity of the F_0_F_1_-ATP synthase complex.

The requirement for F_0_F_1_-ATP synthase complex function in PF cells is intriguing since importance of mt ATP generation by oxidative phosphorylation in PF cells has been questioned [15],[33]. *T. brucei* EATRO1125 PF cells were reported to be 1000 times less sensitive to the F_0_F_1_-ATP synthase inhibitor oligomycin in the presence of glucose than in its absence [57]. In addition, silencing of subunit β of the F_0_F_1_-ATPsynthase led to cell death in glucose-depleted, but not in glucose-rich medium [16]. In contrast, we found that glucose could not rescue the lethal effects of knocking down expression of any of the three subunits tested in our 29–13 PF cells (Figure 4B). Furthermore, the presence of glucose in the medium had little effect on sensitivity of the parental 29–13 cell line to oligomycin (Figure 4C). Finally, while the EATRO1125 PF strain was able to adapt to glucose-depleted medium without a decrease of the doubling time, our PF 29–13 cells grew more slowly after removal of glucose from the medium (doubling time 19.7 h vs. 13.8 h). Thus, the 29–13 PF strain that was used in this study, which is derived from strain 427 [21], appears to differ in its relative glycolytic and oxidative phosphorylation capacity from the EATRO1125 strain that was used in other studies [15],[16]. Moreover, while the bloodstream 1125 cell line is a pleomorphic line and may be more dependent on substrate phosphorylation, the 427 cells are laboratory adapted and unable to establish metacyclic infections in tsetse fly [58],[59]. Thus, they may have lost some of the original metabolic flexibility of procyclic trypanosomes. It would be interesting to test in future studies the dependency on substrate vs. oxidative phosphorylation in recent field isolates of procyclic trypanosomes. Our results, including the fact that mitochondria isolated from RNAi-induced cells had lost the ability to produce ATP via the oxidative phosphorylation pathway suggest that the observed severe growth phenotype after silencing of ATP synthase subunits is caused by inhibition of oxidative phosphorylation and the consequent lack of ATP.

Overall, this study characterized the composition of the F_0_F_1_-ATP synthase complex, identified several protein components that are unique to the *Trypanosoma*, and showed that the conserved α subunit protein and two newly identified subunits, Tb2930 and Tb7760, are important for the structural integrity and proper function of the complex as well as for viability of *T. brucei* PF stage cells. The structural organization of the F_0_F_1_-ATP synthase complex and the specific functions of its protein components remain to be elucidated as does its physiological integration within this organism that regulates oxidative phosphorylation and glycolysis during its life cycle. In conclusion, the identification and characterization of ATP synthase in *T. brucei* represents a major step towards deciphering the unique and essential properties of the respiratory chain of both an early diverged eukaryote and a lethal human parasite.

## Supporting Information

Figure S1 Alignments of ATP synthase subunits delta (A), epsilon (B) and c (C).
(0.02 MB PDF)

Figure S2 Fractionation of TAP_sub b (A) and TAP_sub β (B) TEV eluates on 10–30% glycerol gradients.
(0.92 MB PDF)

Figure S3 MS analysis of *T. brucei* ATP synthase subunit α.
(0.02 MB PDF)

Figure S4 Dot blot analysis of the glycerol gradient-fractionated cleared mitochondrial lysates showing the sedimentation profile of the oxidoreductase complex.
(0.31 MB PDF)

Table S1 LC-MS/MS analysis of *T. brucei* ATP synthase complex.
(0.03 MB XLS)
