# Supplementary material for: The F0F1-ATP Synthase Complex Contains Novel Subunits and Is Essential for Procyclic Trypanosoma brucei
Source: PLoS Pathog. 2009 May 15;5(5):e1000436. doi: 10.1371/journal.ppat.1000436 (PMC2674945; doi:10.1371/journal.ppat.1000436)
Supplement: Figure S1 — Alignments of ATP synthase subunits delta (A), epsilon (B) and c (C). (0.02 MB PDF) [file ppat.1000436.s001.pdf]

SUPPLEMENTARY FIGURE S1

A. Alignment of subunit delta from *Saccharomyces cerevisiae* (S.c., ATP16), *Homo sapiens* (H.s., ATPD) and *Trypanosoma brucei* (T.b., Tb927.6.4990) using CLUSTAL X (1.81) multiple sequence alignment.

```
S.c.            --------MLRSIIGKSASRSLNFVAKRSYAEEAAAASSG----------LKLQFALPHET
H.s.            --------MLPAALLRRPGLGRLVRHARAYAEEAAAPAAASGPNQ----MSFTFASPTQV
T.b.            MFRTFGRRLVSCTLPLLQSAPHDLPEGFEFMEHKVVNKDIHAPHENLETLRLTLTRQDEF
                        :: . :    .    .     : *   ..             : : ::   :

S.c.            LYSGSEVTQVNLPAKSGRIGVLANHVPTVEQLLPGVVEVMEGS-NSKKFFISGGFATVQP
H.s.            FFNGANVRQVDVPTLTGAFGILAAHVPTLQVLRPGLVVVHAEDGTTSKYFVSSGSIAVNA
T.b.            LLREEPVKCVTVTGTNGEYGIYPGHAYKIVQLNPSPLTVEYTDGTTKKYFVSGGFAHINN
                :     *  * :.   .* *: . *. .:  * *. : *   . .:.*:*:*.*  ::

S.c.            DSQLCVTAIEAFPLESFSQENIKNLLAEAKKNVS-SSDAREAAEAAIQVEVLENLQSVLK
H.s.            DSSVQLLAEEAVTLDMLDLGAAKANLEKAQAELVGTADEATRAEIQIRIEANEALVKALE
T.b.            EGSCDVNTVECTLLDDLDLAIAEKELAAQQAALGSAKDDKAKSVVEIRISVIEAVIAALK
                :..  : : *.  *: :.    :  *   :  :  : *    :   *::.. *  :  .*:

S.c.            --
H.s.            --
T.b.            HH
```

B. Alignment of subunit epsilon from *Saccharomyces cerevisiae* (S.c., ATP15), *Homo sapiens* (H.s., ATP5E) and *Trypanosoma brucei* (T.b., Tb10.70.2155) using CLUSTAL X (1.81) multiple sequence alignment.

```
S.c.            ---------MSAWRKAGISYAAYLNVAAQAIRSSLKTELQTASVLNRSQTDAFYTQYKNG
H.s.            ---------VAYWRQAGLSYIRYSQICAKAVRDALKTEFK-ANAEKTSGSNVKIVKVKKE
T.b.            MIRRSCALLSSSWRDHGISYLKYLNVCTETLHSTVKESRR--AKYERWSKPCYTAQRPDG
                          : **. *:**  * ::.:::::.::* .  :     :   .   .:   .

S.c.            TAAS---EPTPITK---
H.s.            -----------------
T.b.            AGGQETIDKVPIHTKDY
```

C. Alignment of three subunits c from *Trypanosoma brucei* using CLUSTAL X (1.81) multiple sequence alignment.

```
Tb927.7.1470      MMRRLAIQSSVRRTTAAITPIAVPMKVASPMCSAAT-RQASTVAISVQGLHYVGTGLAAI
Tb10.70.6340      MMRRLALQSSIRRATPFATPLVASTKALNPMCSAITIREASTVAISVQGLHYVGTGLAAI
Tb11.02.2950      MMRRLALQSSLRRVTPAAVSVMTPAKVTSPIGHAIAIRQASTVAISVQGLHYVGTGLAAI
                  ******:***:**.*.  ..: .. *. .*:  * : *:*********************

Tb927.7.1470      ALAGVGLGIGTIFGNLLVACARQPNLTKMLFNYAILGFALTEAIGLFALMLAFLMLFS
Tb10.70.6340      ALAGVGLGIGTIFGNLLVACARQPNLTKMLFNYAILGFALTEAIGLFALMLAFLMLFS
Tb11.02.2950      ALAGVGLGIGTIFGNLLVACARQPNLTKMLFNYAILGFALTEAIGLFALMLAFLMLFS
                  **********************************************************
```
