# Supplementary material for: The F0F1-ATP Synthase Complex Contains Novel Subunits and Is Essential for Procyclic Trypanosoma brucei
Source: PLoS Pathog. 2009 May 15;5(5):e1000436. doi: 10.1371/journal.ppat.1000436 (PMC2674945; doi:10.1371/journal.ppat.1000436)

## SUPPLEMENTARY FIGURE S2

**S2. Fractionation of TAP_sub *b* (A) and TAP_sub β (B) TEV eluates on 10-30% glycerol gradients.** Fractions were collected from the top of the gradients. Aliquots of odd-numbered fractions were analyzed by SDS-PAGE followed by Sypro Ruby staining. Numbers on the right indicate the position of individual subunits..

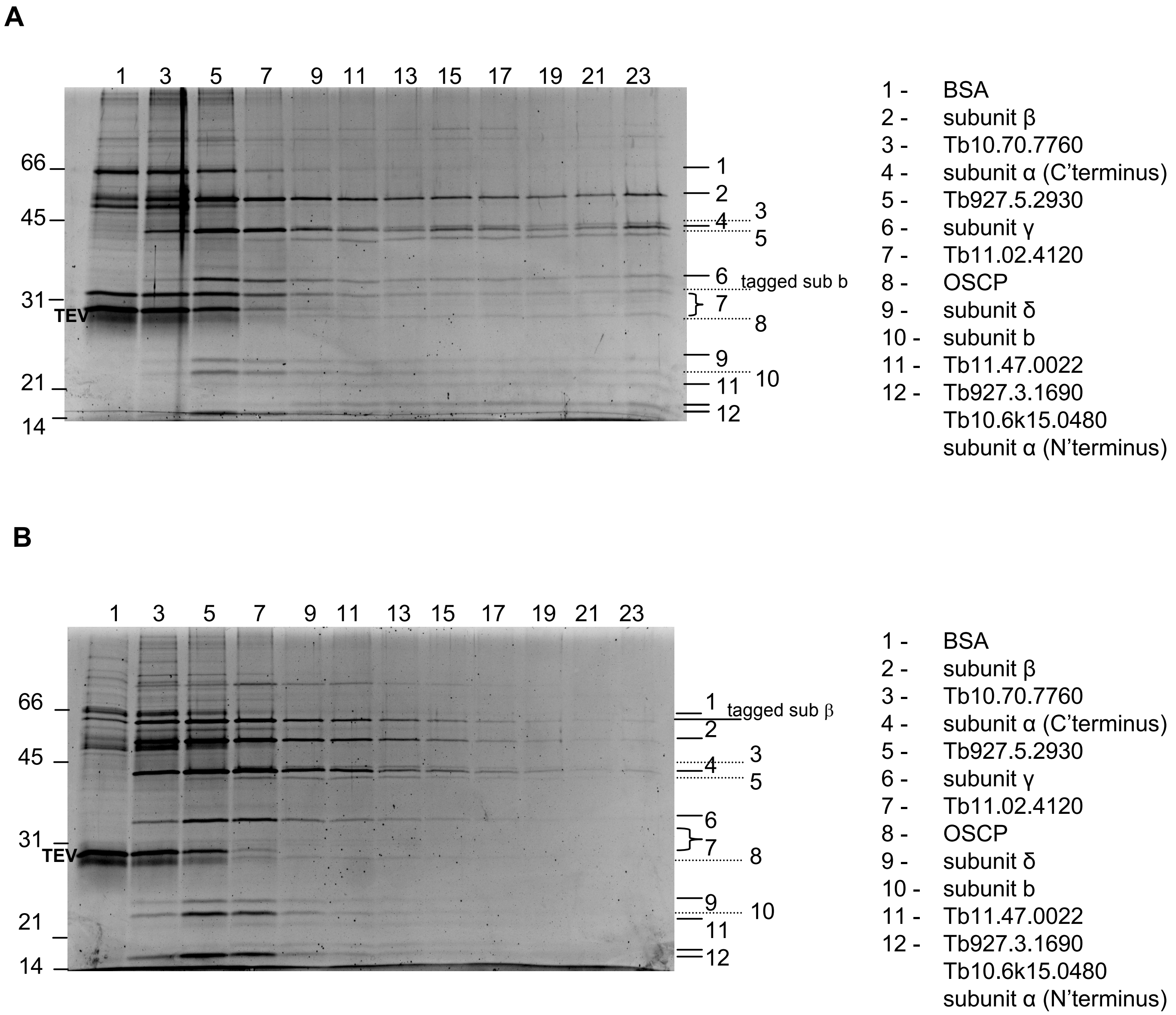

Supplement: Figure S2 — Fractionation of TAP_sub b (A) and TAP_sub β (B) TEV eluates on 10–30% glycerol gradients. (0.92 MB PDF) [file ppat.1000436.s002.pdf]
