# Supplementary material for: The F0F1-ATP Synthase Complex Contains Novel Subunits and Is Essential for Procyclic Trypanosoma brucei
Source: PLoS Pathog. 2009 May 15;5(5):e1000436. doi: 10.1371/journal.ppat.1000436 (PMC2674945; doi:10.1371/journal.ppat.1000436)
Supplement: Figure S3 — MS analysis of T. brucei ATP synthase subunit α. (0.02 MB PDF) [file ppat.1000436.s003.pdf]

SUPPLEMENTARY FIGURE S3

S3. MS analysis of *T. brucei* ATP synthase subunit α.

In red is shown tryptic peptide identified in lower ~14kDa gel band. In blue are shown tryptic peptides in higher ~44kDa gel band. Underlined is sequenced, which was suggested as the site-specific cleavage site characteristic for all trypanosomatid species [36].

>Tb927.7.7420
MRRFGSKFASGLASRCALACPLASAATAPAGASTTSSTSSAQKSFFKTTEMIGYVHSID
GTIATLIPAPGNPGVAYNTIIQIQVSPTTFAAGLVFNLEKDGR**IGIILMDNITEVQSGQ**
KVMATGQLLHIPVGAGVLGKVVNPLGHEVPVGLVTRSRRLL/DSTLGKVDTGAPNIVSR
SPVNYNLLTGFKAVDTMIPIGRGQRELIVGDRQTGK**TSIAVSTIINQVR**INQQILSKNA
VISIYVSIGQRCSNVARIHRLLQSYGALRYTTVMAATAAEPAGLQYLAPYAGVTMGEYF
MNRGRHCLCVYDDLSKQAVAYRQISLLLRRPPGR**EAYPGDVFYLHSR**LLERAAMLSPGK
GGGSVTALPIVETLSNDVTAYIVTNVISITDGQIYLDTKLFTGGQRPAVNIGLSVSRVG
SSAQNAAMKGVAGKLKGILAEYRKLAADSVGGQQVQTIPMIRGAR**FVALFNQK**QPSYFM
NAIVSLYACLNGYLDDVKVQYVKFYEYLLVHRDLGIMYGTAKNKFFYMYVQELNYLIRF
FTLNSPILHGELEEMLKQHTHLFLQHYQSKMNAIKSEKDVKALKNLLYSCKRAV
