# Supplementary material for: The F0F1-ATP Synthase Complex Contains Novel Subunits and Is Essential for Procyclic Trypanosoma brucei
Source: PLoS Pathog. 2009 May 15;5(5):e1000436. doi: 10.1371/journal.ppat.1000436 (PMC2674945; doi:10.1371/journal.ppat.1000436)

SUPPLEMENTARY FIGURE S4

S4. Dot blot analysis of the glycerol gradient-fractionated cleared mitochondrial lysate showing the sedimentation profile of the oxidoreductase complex. Fraction 1 is at the top of the gradient. 100ul aliquots of odd-numbered fractions were analyzed by Dot blot analysis and probed with monoclonal mAb52 antibody.

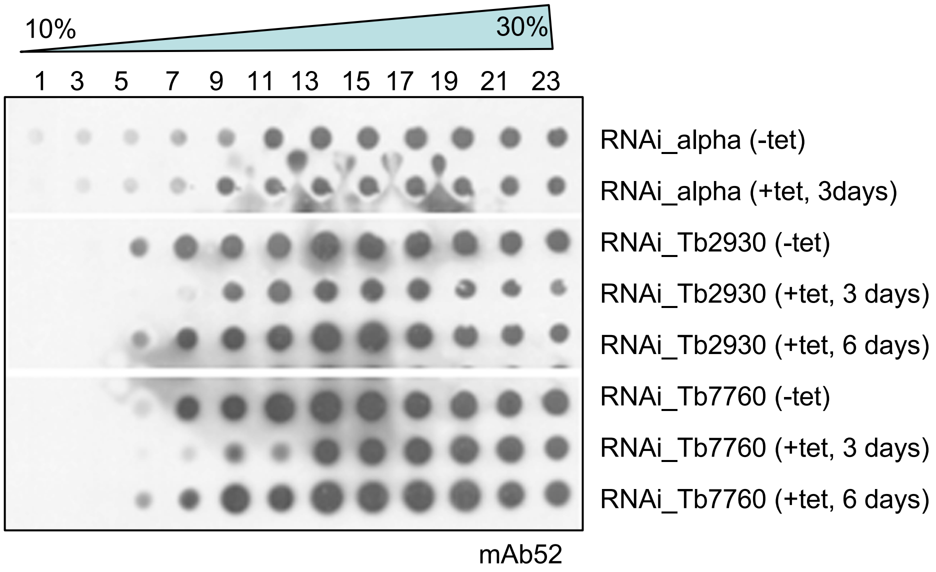

Supplement: Figure S4 — Dot blot analysis of the glycerol gradient-fractionated cleared mitochondrial lysates showing the sedimentation profile of the oxidoreductase complex. (0.31 MB PDF) [file ppat.1000436.s004.pdf]
